# Isolation and Identification of Plant-Growth Inhibitory Constituents from *Polygonum chinense* Linn and Evaluation of Their Bioherbicidal Potential

**DOI:** 10.3390/plants12071577

**Published:** 2023-04-06

**Authors:** Thang Lam Lun, Arihiro Iwasaki, Kiyotake Suenaga, Hisashi Kato-Noguchi

**Affiliations:** 1 Department of Applied Biological Science, Faculty of Agriculture, Kagawa University, Miki 761-0795, Kagawa, Japan; 2 The United Graduate School of Agricultural Sciences, Ehime University, Matsuyama 790-8566, Ehime, Japan; 3 Department of Chemistry, Faculty of Science and Technology, Keio University, Kohoku, Yokohama 223-8522, Kanagawa, Japan

**Keywords:** *Polygonum chinense*, allelopathic substances, (−)-3-hydroxy-*β*-ionone, (−)-3-hydroxy-7,8-*β*-ionone

## Abstract

*Polygonum chinense* Linn. is a medicinal and invasive plant that belongs to the family Polygonaceae. The pharmacological activities and phytochemical constituents of *Polygonum chinense* are well reported, but the allelopathic effects and potent allelopathic substances of *P. chinense* remain to be investigated. Hence, this experiment was conducted to separate and characterize potentially allelopathic substances from an extract of the *Polygonum chinense* plant. The *Polygonum chinense* plant extracts highly suppressed the growth of cress (*Lepidium sativum* L.), lettuce (*Lactuca sativa* L.), barnyard grass (*Echinochloa crusgalli* (L.) P. Beauv.), and timothy grass (*Phleum pratense* L.) seedlings in a species- and concentration-dependent way. Two active substances were separated using a series of purification procedures and determined through spectral analysis as (−)-3-hydroxy-*β*-ionone and (−)-3-hydroxy-7,8-dihydro-*β*-ionone. These two compounds significantly suppressed the seedling growth of *Lepidium sativum* (cress) at concentrations of 0.01 and 1 mM, respectively. The extract concentrations necessary for 50% growth inhibition (*I*_50_ values) of the cress hypocotyls and roots were 0.05 and 0.07 mM for (−)-3-hydroxy-*β*-ionone, respectively, and 0.42 and 1.29 mM for (−)-3-hydroxy-7,8-*β*-ionone, respectively. These findings suggest that these two compounds are in charge of the inhibitory effects of the *Polygonum chinense* extract and may serve as weed control agents.

## 1. Introduction

Alternative weed management strategies based on natural products have received a lot of attention due to the negative effects of synthetic herbicides on agroecosystems. An increase in herbicide-resistant weed species and environmental pollution, including contamination of surface and groundwater bodies, harm to unintended plant species, and negative effects on human health, have all been linked to increased use of intense herbicides [1,2]. To reduce the association between numerous environmental and health concerns and synthetic herbicides, researchers have been looking for alternative sustainable and eco-friendly tools for controlling weeds [3]. One of their most interesting discoveries is the use of allelopathic plant-derived compounds as bioherbicides. Allelopathy is described as the interaction of organisms, including plants and bacteria, that can result in either direct or indirect negative or positive impacts due to the release of chemical substances into the environment [4,5]. Allelochemical processes penetrate the plant rhizosphere through the breakdown of plant leftovers in the soil, rain and fog leaching, root secretion, microbial metabolism, and the release of secondary metabolites into the environment [6,7]. Various types of allelochemicals, such as saponins and alkaloids [8], glycosides, carbohydrates, and amino acids [9], coumaric acid [10], flavonoids and terpenoids [11], and phenols and tannins [12], are produced by plants. These allelochemicals can affect the physiological function of surrounding plants by interrupting respiration, nutrient uptake, enzymatic activities, cell division, and inhibiting cell membrane permeability [13]. The use of allelopathy for non-chemical weed management entails utilizing allelopathic plant varieties, allelopathic cover crops, and allelochemicals as natural herbicides [14]. Compared with synthetic compounds, plant-based natural compounds are an appropriate alternative to synthetic herbicides due to their quick biological disintegration, low chance of weed resistance evolution, and reduced harmfulness to the environment [15], although their efficacy and specificity are unknown or limited [16]. Many plant species, including medicinal plants, can produce and release bioactive compounds that are secondary metabolites into the environment and have the ability to inhibit the growth of other plants.

About three-fourths of the actively biological plant-based compounds currently in widespread use were discovered through follow-up studies to ensure the accuracy of information from folk and traditional medicine usage [17]. There is a crucial characteristic that these fields share: the biological efficacy of a plant extract, whether used for therapeutic purposes or for the prevention of unwelcome plant growth, which is mostly caused by the occurrence of a particular group of secondary products in each plant. In fact, several studies have documented the use of allelopathic plants for managing paddy weeds under field conditions [18,19,20]. According to the results of some researchers, it is agreed that the Dwarf lilyturf (*Ophiopogon japonicus* K.) [21], Mexican sunflower (*Tithonia diversifolia* Hemsl.) [22], houttuynia (*Houttuynia cordata* Thunb.), vetiver grass (*Veteveria zizanioides*) [23], and kava (*Piper methysticum* L.) [24] have inhibitory effects on the selected weeds.

Several researchers have reported that some species of medicinal plants possess allelopathic activity [25,26], and a variety of active substances including novel compounds have been isolated and characterized. Kato-Noguchi et al. [27] reported that two allelopathic substances, (+)-pinoresinol (isolated from *Osmanthus* × *fortune*) and 10-acetoxyligustroside (isolated from *Osmanthus fragrans*) suppressed the growth of *Lolium multiflorum* Lam and *Lepidium sativum* L. (cress). The root and hypocotyl/coleoptile growth of *L. sativum* and *Echinochloa crus-galli* (L.) P. Beauv (barnyard grass) are significantly suppressed by two novel compounds of nimbic acid B and nimbolide B isolated from *Azadirachta indica* (neem) leaves [28] and by two known compounds of 5-hydroxy-3,4-dimethyl-5-pentylfuran-2(5H)-one and 3-hydroxy-*α*-ionone isolated from *Dregea volubilis* leaf extracts [29]. Several have researchers also documented that the root and hypocotyl growth of *L. sativum* is significantly suppressed by loliolide, cis-3-hydroxy-*α*-ionone, and (3R)-3-hydroxy-*β*-ionone isolated from *Elaeocarpus floribundus* Blume [30]; a novel compound of steroidal glycoside 1 and common compound steroidal glycoside 2, isolated from *Marsdenia tenacissima* (Roxb.) Moon leaves extracts [31]; 7,4′,5′-tri-*O*-methylampelopsin and 7,4′,5′-tri-*O*-methyl dihydroquercetin isolated from *Plumbago rosea* [32]; a novel compound of garcienone, isolated from *Garcinia xanthochymus* Hook [33]. In addition, four characterized allelochemicals of (−)-catechin, (−)-epicatechin, resveratroloside, and piceatannol glucoside were extracted from *Polygonum cuspidatum* Sieb. and Zucc., which belongs to the family of Polygonaceae, and significantly inhibited *Lepidium sativum* L. [34].

The Polygonaceae family, which consists of 49 genera and over 1200 species, is a type of predominantly herbaceous plant with a worldwide distribution [35]. Notable members of Polygonaceae include many noisome and invasive weeds, and several species are used in traditional medicine [36,37]. *Polygonum chinense* Linn. is a species that belongs to the family of Polygonaceae and is also known as smartweed. This species is distributed mostly in north-temperate climates such as the Philippines, India, Myanmar, Thailand, Japan, Bhutan, Vietnam, Indonesia, Malaysia, Sikkim, and Nepal [36,38]. *Polygonum chinense* is a climbing perennial plant that produces much-branched stems from stout, underground rhizomes. The stems can be 0.3–6 m long and often become woody at the base. At higher elevations, the plant is often small and erect [39]. They are harvested from the wild for local use as food and traditional medicine to treat chest diseases, fever, whooping cough, and wounds [40]. A paste made from its leaves is used as an external application for boiling. Juice made from the stem is consumed internally as a tonic, a vulnerary, and to treat fevers.

According to extensive research, *Polygonum chinense* possesses a variety of pharmacological characteristics such as anti-bacterial, anti-fungal, antioxidant [41], anti-inflammatory [42], hepatoprotective [42], anti-tumor [43], anti-diarrheal [44], gastroprotective [45], and cytotoxic, antioxidant, and anti-microbial characteristics [40]. Phytochemical analysis was performed on the plant extracts using standard techniques to identify secondary metabolites such as amino acids, glycosides and carbohydrates, fats and fixed oils, flavanones and flavones, mucilage and gums, tannins, phenolic compounds, sterols, proteins, saponins, alkaloids, and triterpenoids [41,46]. Despite the numerous studies on the pharmacological properties and phytochemical constituents of *P. chinense*, the allelopathic potential of *P. chinense* plant extracts has not yet been investigated. Consequently, the current study aimed to evaluate the allelopathic activities of *P. chinense* and to determine the allelopathic substances that can be used as potential candidates for bioherbicides.

## 2. Results

### 2.1. Growth Inhibitory of Polygonum chinense Plant Extracts

**Figure 1:**
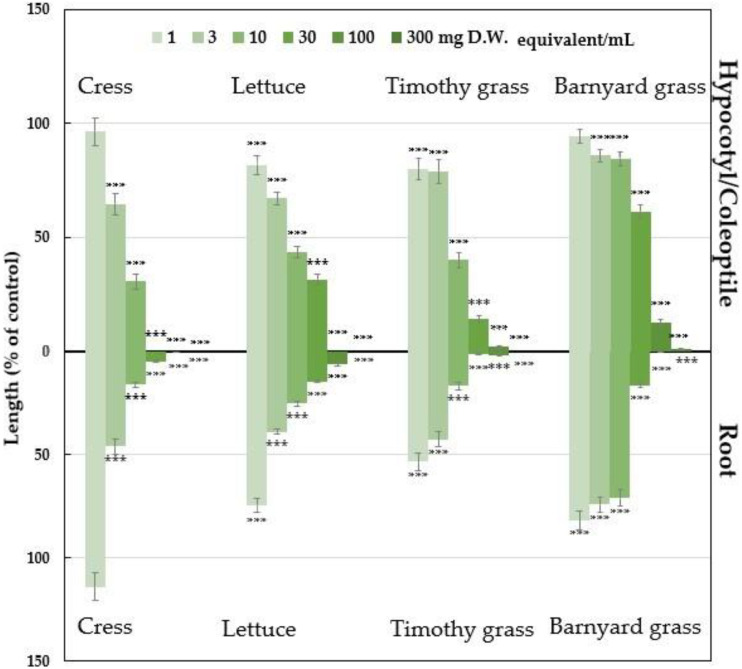
Effect of *Polygonum chinense* plant extracts treated with different concentrations of 0.001, 0.003, 0.01, 0.03, 0.1, and 0.3 mg D.W. equivalent extract/mL (D.W.: dry weight) on the hypocotyls/coleoptiles and the root length of four test plant species. The means ± SE from six replications with 10 seedlings per replication (n = 60) for each determination are shown. *** represents a statistically significant difference between the control and the treatment: *p* < 0.001 (post hoc Tukey’s test).

**Figure 2:**
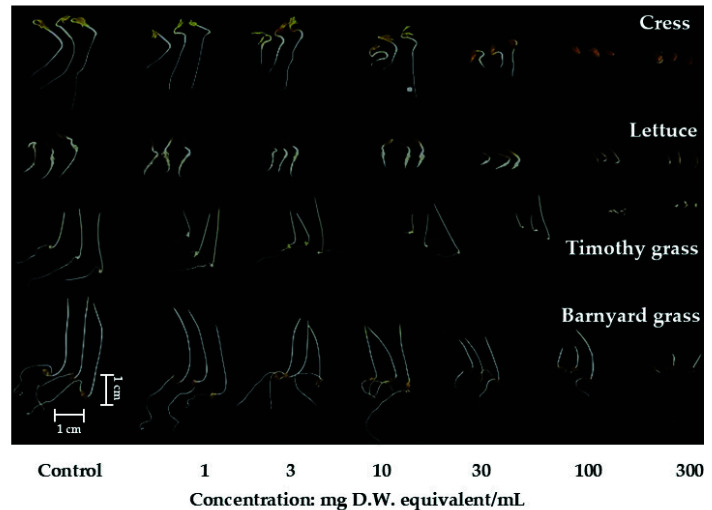
Effect of *Polygonum chinense* plant extracts on the hypocotyls/coleoptiles and root length of four test plant species at different extract concentrations after 48 h of treatment. (D.W.: dry weight).

The aqueous methanol extracts of Polygonum chinense showed different inhibiting activities depending on the concentration of the extract and test plant species (Figure 1 and Figure 2; Tables S1 and S2). Inhibition of seedling growth by the plant extracts began from the lowest dose of 1 mg D.W. equivalent extract/mL, while the cress seedlings and barnyard grass coleoptiles began from 3 mg D.W. equivalent extract/mL. At the highest dose of 300 mg D.W. equivalent extract/mL, the growth of the tested seedlings was entirely inhibited, apart from the coleoptile growth of barnyard grass (0.91%) compared with the control. Significant inhibition of greater than 50% was found for the hypocotyl growth of cress and lettuce and the coleoptile growth of timothy grass at 10 mg D.W. equivalent extract/mL (inhibited to 30.54, 43.42, and 40% of the control, respectively) and for barnyard grass at 100 mg D.W. equivalent extract/mL (12.5% of the control) (Table S1). Significant inhibition of greater than 50% was found for the root growth of cress, lettuce, and timothy grass and was inhibited to 46, 39.01, and 42.48% of the control at 3 mg D.W. equivalent extract/mL, respectively, and barnyard grass to 16.26% of the control at 30 mg D.W. equivalent extract/mL (Table S2).

The concentration needed to suppress 50% (*I*_50_ values) of the hypocotyls/coleoptiles and root growth was 5.01–35.09 mg D.W. equivalent extract/mL and 2.54–11.70 mg D.W. equivalent extract/ mL, respectively (Table 1). Comparing the *I*_50_ values for the hypocotyls/coleoptiles and root growth, the roots of all the tested plants were more responsive to the plant extracts than their hypocotyls/coleoptiles. According to the correlation coefficient (R), the hypocotyls/coleoptiles and root length of each test plant were significantly negatively correlated with the concentration of the *P. chinense* extracts (*p* < 0.01) (Table 1).

### 2.2. Isolation and Characterization of the Allelopathic Substances from the Polygonum chinense Extracts

**Figure 3:**
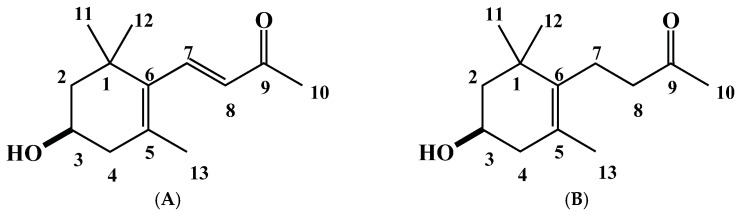
The chemical structures of the characterized allelopathic substances from *Polygonum chinense* extracts of Compound **1**, (−)-3-hydroxy-*β*-ionone (**A**) and Compound **2,** (−)-3-hydroxy-7,8-dihydro-*β*-ionone (**B**).

Compound **1**, the molecular formula of C_13_H_20_O_2_, was established through HRESIMS at *m*/*z* 209.1534 [M+H]^+^ (calcd for C_13_H_21_O_2_, 209.1542). The spectrum data of ^1^H NMR (400 MHz, CDCl_3_) spectrum of the compound showed δ_H_ 7.21 (d, J = 16.9 Hz, 1H, H-7), 6.11 (d, J = 16.9 Hz, 1H, H-8), 2.08 (dd, J = 17.2, 9.7 Hz, 1H, H-4), 2.43 (dd, J = 17.2, 5.4 Hz, 1H, H-4), 1.49 (m, 1H, H-2), 1.79 (m, 1H, H-2), 4.01 (m, 1H, H-3), 1.12 (s, 3H, H-11), 1.11 (s, 3H, H-12), 2.30 (s, 3H, H-13). The specific rotation of the compound was [α]_D_^27^ −52 (*c* = 0.065, CHCl_3_). Based on ^1^H NMR spectra data and compared with previously reported data, the compound was identified as (−)-3-hydroxy-*β*-ionone (compound **1**, Figure 3A) [47,48].

Compound **2**, the molecular formula of C_13_H_22_O_2_, was established through HRESIMS at *m*/*z* 211.1683 [M+H]^+^ (calcd for C_13_H_23_O_2_, 211.1693). The spectrum data of ^1^H NMR (400 MHz, CDCl_3_) showed δ_H_ 3.93 (m, 1H, H-3), 2.52–2.46 (m, 2H, H-8), 2.35–2.15 (m, 3H, H-7, H-4a), 2.14 (s, 3H, H-10), 1.95 (dd, J = 16.3, 10.0 Hz, 1H, H-4b), 1.71 (m, 1H, H-2a), 1.59 (s, 3H, H-13), 1.42 (dd, J = 11.8 Hz, 1H, H-2b), 1.03 (s, 3H, H-11), 1.02 (s, 3H, H-12). The carbon NMR spectrum 100 MHz, with CDCl_3_ as the internal standard, showed δc 208.9 (C-9), 136.1 (C-6), 125.1 (C-5), 65.3 (C-3), 48.6 (C-2), 44.4 (C-4), 42.3 (C-8), 38.0 (C-1), 30.0 (C-12), 29.6 (C-12), 28.5 (C-11), 22.0 (C-7), 19.8 (C-13). The specific rotation of the compound was [α]_D_^27^ −48 (*c* = 0.090, CDCl_3_). Based on ^1^H NMR spectra data and comparing them with earlier published data, the compound was identified as (−)-3-hydroxy-7,8-dihydro-*β*-ionone (compound **2**, Figure 3B) [49,50].

### 2.3. Growth Inhibitory Effects of the Two Compounds

**Figure 4:**
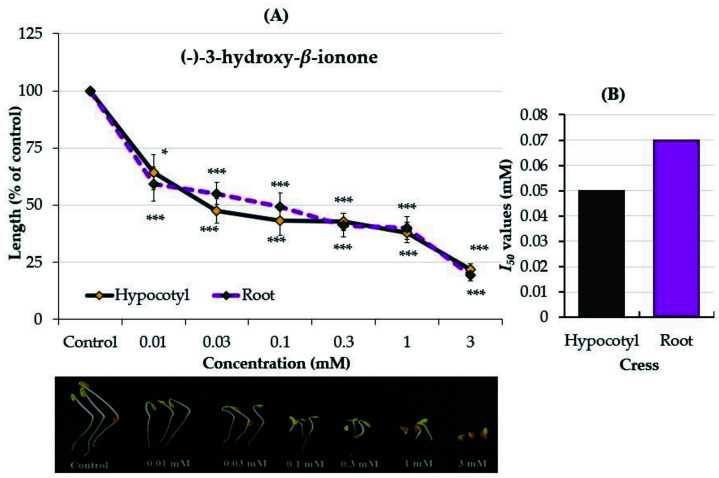
(**A**) Effects of (−)-3-hydroxy-*β*-ionone on the seedling growth of cress (*Lepidium sativum)* and (**B**) *I*_50_ values. The means ± SE from three replications with 10 seedlings per replication (n = 30) for each determination are shown. *, and *** represent statistically significant differences between the control and the treatment: *p* < 0.05, *p* < 0.001 (post hoc Tukey’s test).

**Figure 5:**
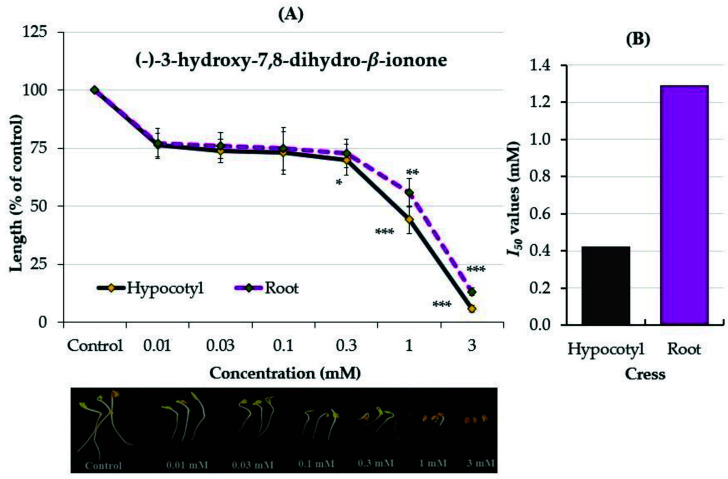
(**A**) Effects of (−)-3-hydroxy-7,8-dihydro-*β*-ionone on the seedling growth of cress (*Lepidium sativum)* and (**B**) *I*_50_ values. The means ± SE from 3 replications with 10 seedlings per replication (n = 30) for each determination are shown. *, **, and *** represent a significant difference between the control and the treatment: *p* < 0.05, *p* < 0.01, *p* < 0.001 (post hoc Tukey’s test).

The identified compounds of (−)-3-hydroxy-*β*-ionone (compound **1**) and (−)-3-hydroxy-7,8-dihydro-*β*-ionone (compound **2**) were assayed against cress to confirm their allelopathic properties. Both compounds significantly inhibited the cress growth compared with the control (*p* < 0.05, Figure 4 and Figure 5). Compound **1** significantly inhibited the hypocotyl and root growth of cress to 64.3 and 59.1% of the control at the concentration of 0.01 mM while compound **2** significantly inhibited the hypocotyls to 70% of the control at 0.3 mM and the roots to 55% of the control at 1 mM. At the highest concentration of 3 mM, the two compounds suppressed the hypocotyl and root growth of the cress by more than 75%.

The *I*_50_ values of compound **1** for the hypocotyl and root growth of cress were 0.05 and 0.07 mM (Figure 4B), respectively, and for compound **2**, they were 0.42 and 1.29 mM (Figure 5B), respectively. When comparing the two compounds based on the results of the *I*_50_ values, compound **1** had a greater inhibitory effect on the seedling growth of cress than compound **2**.

## 3. Discussion

The *Polygonum chinense* aqueous methanol extracts had growth-suppression effects on the four tested plants. The effectiveness of the plant extracts relies on the extract concentrations and tested plant species. The species- and concentration-dependent inhibitory effects are also consistent with many research findings on different plant extracts of *Leucas cephalotes* (Roth) Spreng [51], *Annona muricana* (L.) [52], *Senna garrettiana* [53], *Dregea volubilis* (L.f.) [29], *Elaeocarpus floribundus* Blume [30], *Marsdenia tenacissima* (Roxb.) Moon [31], and *Plumbago rosea* [32]. In addition, the *I*_50_ values revealed that the roots of the four tested plants were more susceptible to extracts of *P. chinense* than the hypocotyls/ coleoptiles. Similar results were also obtained by Khan and Kato-Noguchi [54] and Krumsri et al. [55]. The involvement of both cell expansion and cell proliferation in root growth causes high sensitivity in the roots to allelopathic substances [56,57]. The permeability of allelopathic substances is higher in root surfaces than in the hypocotyl/coleoptile [58]. These findings have indicated that the growth-suppression impact might be due to active substances in the plant extracts.

Bioassay-guided separation and purification of the *P. chinense* extract through a series of reversed-phase columns led to the isolation of two active inhibitory substances which have been described as (−)-3-hydroxy-*β*-ionone (compound **1**, Figure 3A) and (−)-3-hydroxy-7,8-dihydro-*β*-ionone (compound **2**, Figure 3B). Both compounds are C_13_-norisoprenoid aglycons, which are typically seen to be descendent from carotenoids through oxidative degradation [59]. (−)-3-Hydroxy-*β*-ionone has been documented as a bound constituent of several fruit tissues, such as apple [60], grape [61], and papaya [62]. Compound **1** accumulates in the seedlings of bean varieties through irradiation by light, causing light-induced growth inhibition of bean seedlings [63]. (−)-3-Hydroxy-*β*-ionone has also been isolated and identified from various plants, and its growth inhibition potential against a number of species is well reported [30,47,64,65,66]. Aloum et al. [67] also reported that (−)-3-hydroxy-*β*-ionone retards the colony formation, proliferation, and cell migration of human squamous cell carcinoma. (−)-3-Hydroxy-7,8-dihydro-*β*-ionone has already been described as a conjugate in the aqueous extract of *Epimedium grandiflorum* var. *thunbergianum* [68]. It has also been isolated from *Chamaecyparis formosensis* [50]. Although (−)-3-hydroxy-*β*-ionone and (−)-3-hydroxy-7,8-dihydro-*β*-ionone have been documented in many plants, there have been no reports from *Polygonum chinense*. Therefore, this study is the first to document the presence of the (−)-3-hydroxy-*β*-ionone and (−)-3-hydroxy-7,8-dihydro-*β*-ionone compounds in *Polygonum chinense* extracts and their potentially allelopathic activity.

The results of the present study showed that (−)-3-hydroxy-*β*-ionone and (−)-3-hydroxy-7,8-dihydro-*β*-ionone have a significant inhibitory effect against the growth of cress seedlings (Figure 4 and Figure 5). Both compounds have similar structures with different functional groups. In (−)-3-hydroxy-*β*-ionone, the C-7 and C-8 positions are linked with the carbon–carbon double bond (olefins carbon) functional group, while (−)-3-hydroxy-7,8-dihydro-*β*-ionone is linked with a carbon–carbon single bond in the functional group. The *I*_50_ values indicated that the cress seedlings were more sensitive to (−)-3-hydroxy-*β*-ionone compared with (−)-3-hydroxy-7,8-dihydro-*β*-ionone at lower concentrations (Figure 4 and Figure 5). The presence of the olefin carbon functional group may be the reason for their different allelopathic activities. Allelopathic compounds vary in their mode of action, uptake, and effectiveness [5,14]. These findings indicate that both substances possess potentially allelopathic properties and may make an important contribution to the allelopathic substances of *Polygonum chinense*. Hence, *Polygonum chinense* might be useful in weed management through the application of its extracts, the inclusion of its residues or different parts as mulch, and the application of its active substances as natural product-based agriculture to reduce synthetic chemical herbicides usage and also to attain sustainable crop production for pollutant-free green environments. Therefore, this information might be useful as a way to study the interrelations between active compounds and action targets in the target plants which leads to suitably applying them in fieldwork. However, further field experiments are required to clarify and confirm the allelopathic potency of *P. chinense.*

## 4. Materials and Methods

### 4.1. Plant Materials

*Polygonum chinense* was gathered from different villages of Mandalay Division, Myanmar, from July–August 2020 (Figure S1). The collected plant materials were washed and dried in the shade. The dried plant samples were then ground into a fine powder and stored at 2 °C in a vacuum-sealed plastic package for extraction. The seeds of two dicotyledons [*Lepidium sativum* L. (cress) and *Lactuca sativa* L. (lettuce)] and two monocotyledons [*Echinochloa crusgalli* (L.) P. Beauv. (barnyard grass) and *Phleum pratense* L. (timothy grass)] were chosen for testing the allelopathic potential of the extracts. The dicot seeds of *L. sativa* and *L. sativum* were bought from Nakahara Seed Product Co., Ltd., Fukuoka, Japan, and Mikado Kyowa Seed Co., Ltd., Chiba, Japan, respectively. The monocot seeds of *E. crusgalli* were procured from a farmer’s field in Miki, Japan, and the seeds of *P. pratense* were obtained from Snow Brand Seed Co., Ltd. (Sapporo, Japan).

### 4.2. Extraction and Growth Inhibition Assay of P. chinense

Preliminary extraction was carried out with 50 g of *Polygonum chinense* plant powder which was soaked with 300 mL of 70:30 (*v*/*v*) methanol: distilled water for 48 h and then filtrated through a Buchner funnel with a 125 mm (No. 2) filter paper layer (Advantec, Toyo Roshi Kaisha Ltd., Tokyo, Japan). The leftovers were immediately re-soaked for 24 h in 300 mL of methanol before being filtrated once again. These two filtrates were combined and dried using a rotavapor (Yamato Scientific Co., Ltd., Tokyo, Japan) set to 40 °C. The crude extracts were diluted with 100 mL of MeOH to prepare the desired six assay concentrations (i.e., 1, 3, 10, 30, 100, and 300 mg dry weight (D.W.) equivalent extract/mL) and controls (0.6 mL of a 0.05% (*v*/*v*) aqueous solution of Tween 20 (polyoxyethylene sorbitan monolaurate; Nacalai Tesque, Inc., Kyoto, Japan). The desired concentration amount of the extract was added to 28 mm-diameter (No. 2) filter papers (Toyo Roshi Ltd., Tokyo, Japan) in Petri dishes (diameter 28 mm) and dried in a draft chamber. After drying the extract, each Petri dish was soaked with 0.6 mL of a 0.05% (*v*/*v*) aqueous Tween 20 solution. In addition, the Petri dishes were added to with an aqueous Tween 20 solution (0.6 mL of a 0.05% (*v*/*v*)) without the extracts of *P. chinense* as a control treatment. Ten monocots (barnyard grass and timothy grass) seeds (sprouted, 36 h at 25 °C in the dark) and ten dicots (cress and lettuce) seeds were added to each Petri dish. All the Petri dishes were put into a tray and then enclosed with polyethylene film and aluminum foil and stored in a growth chamber for 48 h in the dark at 25 °C. A ruler was used to measure the lengths of the hypocotyls/coleoptiles and the roots. The experiment was carried out by CRD (completely randomized design) with six replications of the six concentrations and the control (10 seeds/sprouted seeds for each concentration). Inhibition of the root and hypocotyl/coleoptile growth was calculated by comparing them with the control. The following formula was used to compute the inhibition % of seedling growth:

$$(\%)\;\mathrm{seedling}\;\mathrm{growth}=1-\frac{length\;of\;treated\;seedlings}{length\;of\;control\;seedlings}\times 100$$

### 4.3. Separation and Isolation of Active Substances from the Polygonum chinense Extracts

The extraction procedure for the *Polygonum chinense* plant powder (2.1 kg) was performed as stated therein (4.2). To produce a concentrated extract, the obtained extracts were then desiccated by a rotavapor (40 °C), and the concentrated extract was then suspended in distilled water. The solvent was adjusted to a pH of 7.0 with the buffer of 1 M phosphate followed by partitioning four times with the same volume of ethyl acetate to separate the aqueous and ethyl acetate portions. After being soaked with anhydrous Na_2_SO_4_ for the entire night, the ethyl acetate fraction was filtered and evaporated until dry. The fraction of ethyl acetate was selected for further isolation and purification step.

The extract was separated by chromatography using 60 g of silica gel (70–230 mesh, silica gel 60; Nacalai Tesque) and eluted stepwise of *n*-hexane: ethyl acetate into 9 fractions as 120:30, 105:45, 90:60, 75:75, 60:90, 45:105, 30:120 (*v*/*v*; 150 mL/ step), 150 mL of ethyl acetate, and using 300 mL of methanol for the last fraction. The cress seeds were used to determine the allelopathic activity, as described in (4.2). The results of the cress assay data showed that the most active potential was eluted in 45:105 *n*-hexane: ethyl acetate fraction (F_6_). F_6_ was then evaporated to dryness and subjected to Sephadex LH-20 chromatography (100 g; GE Healthcare, Uppsala, Sweden). A mobile phase was used as methanol in distilled water with 20, 40, 60, and 80% (20% per step *v*/*v*, 150 mL) and 300 mL of methanol. The active fraction (40% methanol in distilled water F_2_) obtained from Sephadex LH-20 was separated using a reverse-phase C_18_ cartridge (1.2 × 6.5 cm; YMC Co. Ltd., Kyoto, Japan). The cartridge was loaded with 20, 30, 40, 50, 60, 70, 80, and 90% methanol in distilled water (10% per step, *v*/*v*, 15 mL) and 30 mL of methanol was used for the final fraction.

The most active fraction was obtained from 40% (F_2_) methanol in distilled water. F_2_ was then purified and run at a flow rate of 1.5 mL/min with 45% (*v*/*v*) methanol in distilled water and detected at 220 nm using reverse-phase high-performance liquid chromatography (HPLC column, 500 × 10 mm I.D., ODS AQ-325; YMC Ltd., Shimadzu Corporation, Kyoto, Japan). The two most active fractions were detected at the retention times of 153–158 and 167–175 min. The active fractions were then purified again and run at a flow rate of 0.5 mL/min with 35% (*v*/*v*) methanol in distilled water and detected at 220 nm of the retention times 90–94 min (compound **1**, Figure 3A) and 100–104 min (compound **2**, Figure 3B). using a reverse-phase HPLC column (250 × 4.6 mm I.D., Inertsil ODS-3; GL Science Inc., Tokyo, Japan). Last, the chemical structures of the two substances were identified by the analyses of the HRESIMS and ^1^HNMR spectra (400 MHz, CDCl_3_) and the optical rotation.

### 4.4. Germination Test for the Compounds

The identified compounds were dissolved in 1 mL of methanol and prepared in 28 mm Petri dishes containing filter paper to obtain the 5 desired assay concentrations of 0.03, 0.1, 0.3, 1, and 3 mM. After the filter papers were completely dry, they were treated with 0.6 mL of 0.05% (*v*/*v*) distilled water (aqueous) Tween 20 solution. The germination test was carried out using a completely randomized design with three replications (10 seedlings/replication). A total of ten uniform cress seeds were tested in each Petri dish and kept for 48 h in the darkness at 25 °C under a growth chamber. The hypocotyl and root lengths of the cress seedlings were measured with a ruler after 48 h of growth. Data collection was carried out and was contrasted with the seedlings used as the control.

### 4.5. Statistical Analysis

The growth assays were arranged with three replications using CRD (completely randomized design) and the whole assay was duplicated. All recorded data were presented with means ± standard error. The Statistical Package for the Social Sciences (SPSS, version 16.0) was used to analyze the one-way analysis of variance (ANOVA). Significant differences among the treatments and control were analyzed using a post hoc Tukey’s test at *p* < 0.05, *p* < 0.01, and *p* < 0.001. The effective concentration required to suppress 50% (*I*_50_ values) for each test plant was calculated by GraphPad Prism 6.0.

## 5. Conclusions

The *Polygonum chinense* aqueous methanol plant extracts suppressed the hypocotyl/ coleoptile and root growth of the tested plant species, i.e., cress (*Lepidium sativum* L.), lettuce (*Lactuca sativa* L.), barnyard grass (*Echinochloa crusgalli* (L.) P. Beauv.), and timothy grass (*Phleum pratense* L.). The degree of growth suppression varied with the level of extract concentration and the tested plant species. Two allelopathic substances, (−)-3-hydroxy-*β*-ionone and (−)-3-hydroxy-7,8-dihydro-*β*-ionone, were identified, and they showed inhibitory effects against cress. This study indicates that the plant residue of *Polygonum chinense*, its plant extracts, and its active substances could be used as natural sources of bioherbicide. Further studies are needed to elucidate its role in plant–plant interactions in natural environments and to explore whether adding adjuvants in preparing formulations can further enhance its herbicidal activity.

## Figures and Tables

**Table 1:** *I*_50_ values (concentration of *Polygonum chinense* extracts required to suppress 50% of seedling growth), and the correlation coefficient (R) between the concentration of the *P. chinense* extracts and the hypocotyls/coleoptiles and root growth of the four tested plants.

Test Plants	*I*_50_ Value (mg D.W. Equivalent Extract/mL)		Correlation Coefficient (R)	
	Hypocotyl/ Coleoptile	Root	Hypocotyl/ Coleoptile	Root
Cress	5.01	2.97	−0.789 **	−0.744 **
Lettuce	7.25	2.54	−0.852 **	−0.864 **
Timothy grass	7.00	1.46	−0.803 **	−0.796 **
Barnyard grass	35.09	11.70	−0.832 **	−0.805 **

** Correlation (R) designates significant variations at the 0.01 level (two-tailed Pearson’s correlation).

## Data Availability

Not applicable.

## Supplementary Materials

The following supporting information can be downloaded at: https://www.mdpi.com/article/10.3390/plants12071577/s1. Figure S1: *Polygonum chinense* (source: the photos were taken by Thang Lam Lun (July 2020) near Mandalay division, Myanmar); Table S1: Mean values of the *Polygonum chinense* plant extracts on the hypocotyls/coleoptiles length of four test plant species at different extract concentrations after 48 h of treatment; Table S2: Mean values of the *Polygonum chinense* plant extracts on the roots length of four test plant species at different extract concentrations after 48 h of treatment.
