# Supplementary material for: Isolation and Identification of Plant-Growth Inhibitory Constituents from Polygonum chinense Linn and Evaluation of Their Bioherbicidal Potential
Source: Plants (Basel). 2023 Apr 6;12(7):1577. doi: 10.3390/plants12071577 (PMC10096564; doi:10.3390/plants12071577)
Supplement: Supplementary file 1 [file plants-12-01577-s001.zip › plants-2292127-supplementary.pdf]

Supplementary

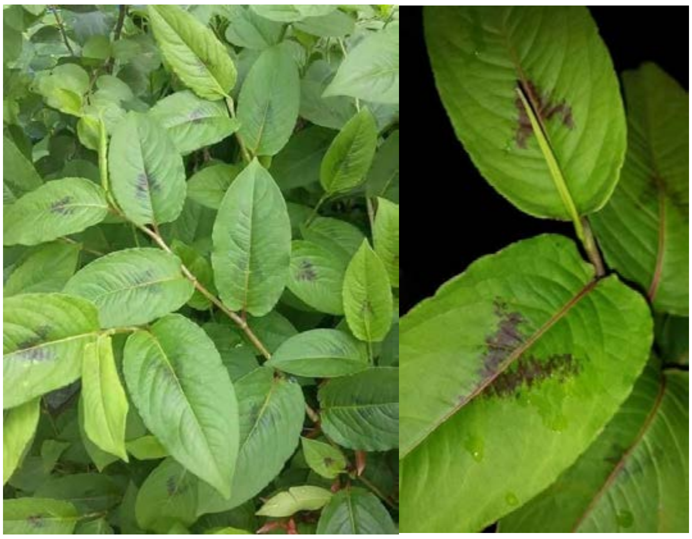

Figure S1. *Polygonum chinense* (Source: the photos were taken by Thang Lam Lun (in July 2020) near Mandalay division, Myanmar)

Table S1: Mean values of *Polygonum chinense* plant extracts on the hypocotyls/ coleoptiles length of four test plant species at different extract concentrations after 48 h treatments.

Concentrations (mg dry weight equivalent/mL)	Test Plants (% of Hypocotyls/ coleoptiles)			
	Cress	Lettuce	Timothy	Barnyard grass
Control	100	100	100	100
1	96.41	81.58***	80.00***	94.40
3	64.67***	67.11***	78.82***	85.81***
10	30.54***	43.42***	40.00***	84.38***
30	0.00***	31.58***	14.12***	61.33***
100	0.00***	0.00***	1.96***	12.50***
300	0.00***	0.00***	0.00***	0.91***

Asterisks represent a statistically significant difference between treatment and control: ***$p < 0.001$ (one-way ANOVA, post hoc by Tukey's test)

Table S2: Mean values of *Polygonum chinense* plant extracts on the root length of four test plant species at different extract concentrations after 48 h treatments.

Concentrations (mg dry weight equivalent/mL)	Test Plants (% of Root)			
	Cress	Lettuce	Timothy	Barnyard grass
Control	100	100	100	100
1	113.96	74.44***	53.27***	81.77***
3	46.00***	39.01***	42.48***	74.06***
10	16.02***	25.11***	16.67***	70.94***
30	4.58***	14.35***	1.31***	16.26***
100	0.00***	5.83***	1.63***	0.00***
300	0.00***	0.00***	0.00***	0.00***

Asterisks represent a statistically significant difference between treatment and control: ***$p < 0.001$ (one-way ANOVA, post hoc by Tukey's test)
